# Hypertensive Disorders of Pregnancy and Fetal Growth Restriction: Clinical Characteristics and Placental Lesions and Possible Preventive Nutritional Targets

**DOI:** 10.3390/nu14163276

**Published:** 2022-08-10

**Authors:** Daniela Denis Di Martino, Laura Avagliano, Enrico Ferrazzi, Federica Fusè, Vittoria Sterpi, Marco Parasiliti, Tamara Stampalija, Sara Zullino, Antonio Farina, Gaetano Pietro Bulfamante, Matteo Di Maso, Francesco D’Ambrosi

**Affiliations:** 1 Department of Woman, Child and Neonate, Fondazione IRCCS Ca’ Granda Ospedale Maggiore Policlinico, 20122 Milan, Italy; 2 Department of Health Sciences, San Paolo Hospital, ASST Santi Paolo e Carlo, 20142 Milano, Italy; 3 Department of Clinical and Community Health Sciences, University of Milan, 20122 Milan, Italy; 4 Department of Woman, Mother and Neonate, Buzzi Children’s Hospital, 20154 Milan, Italy; 5 Unit of Fetal Medicine and Prenatal Diagnosis, Institute for Maternal and Child Health, IRCCS Burlo Garofolo, 34137 Trieste, Italy; 6 Department of Medicine, Surgery and Health Sciences, University of Trieste, 34127 Trieste, Italy; 7 Department of Obstetrics and Gynaecology, Pisan University Hospital, 56124 Pisa, Italy; 8 Division of Obstetrics and Prenatal Medicine, Department of Medicine and Surgery (DIMEC), Sant’Orsola-Malpighi Hospital, University of Bologna, 40126 Bologna, Italy; 9 Unit of Human Pathology, San Paolo Hospital, ASST Santi Paolo e Carlo, 20142 Milan, Italy; 10 Department of Clinical Sciences and Community Health, Branch of Medical Statistics, Biometry and Epidemiology “G.A. Maccacaro”, University of Milan, 20122 Milan, Italy

**Keywords:** fetal vascular malperfusion, feto-placental Doppler, maternal vascular malperfusion, Mediterranean diet, placenta pathology, preeclampsia, sFlt-1/PlGF ratio

## Abstract

Background: The purpose of this study was to describe the placental lesions in pregnancies complicated by hypertensive disorders (HDP) and/or fetal growth restriction (FGR) and in uneventful control pregnancies. Methods: This is a case control study that included singleton pregnancies with HDP and normally grown fetus (HDP-AGA fetus), with HDP and FGR, early FGR, late FGR, and uneventful pregnancies. Feto-placental Doppler velocimetry and sFlt-1/PlGF ratio were performed. Placental histology was evaluated blinded according to the Amsterdam Consensus criteria. Results: Placental lesions with maternal vascular malperfusion (MVM) were significantly more frequent in HDP-FGR and early FGR (92% and 83%). MVM were significantly associated with abnormal feto-placental Doppler parameters, especially in early FGR. Delayed villous maturation (DVM) was associated with late FGR (83%). HDP-AGA fetus cases presented a heterogeneous pattern of placental lesions, including 60% of cases with MVM, but were not associated with abnormal Doppler feto-placental velocimetry. Conclusions: We found a prevalence of placental maternal vascular malperfusion in HDP-FGR and early FGR groups. These lesions were also associated with abnormal, anti-, and angiogenic markers. Conversely HDP-AGA fetus and late FGR presented more heterogeneous placental lesions not severe enough to cause feto-placental Doppler anomalies. These conditions are likely associated with different etiologies, such as maternal pre-pregnancy risk factors for metabolic syndrome. These findings suggest a possible preventive nutritional approach in addition to low-dose aspirin in pregnant women with predisposing factors for HDP-AGA fetuses and late FGR.

## 1. Introduction

Hypertensive disorders of pregnancy (HDP) and fetal growth restriction (FGR) are among the most relevant obstetrical syndromes associated with maternal and/or fetal morbidity and mortality [1]. Among the different hypertensive disorders, even preeclampsia (PE), possibly the most severe form of HDP, is widely acknowledged as a syndrome [2] rather than as a single disease occurring with different complications at different gestational ages. Indeed, various clinical-pathogenetic phenotypes of HDP have been proposed: on one side, early shallow trophoblastic invasion with poor placental and fetal growth and, on the other side, maternal “cardiovascular and metabolic risk factors for endothelial dysfunction” [3], both leading to oxidative stress [4,5].

In the placenta, the exchange of nutrients and oxygen between mother and fetus occurs at the interface of the placental villi with their vasculosyncytial membranes and the intervillous space in contact with the maternal blood [6]. In physiological pregnancy, during implantation, there is an invasion of extravilli trophoblasts in the maternal decidua and spiral arteries with consequent remodeling and formation of a low-resistance circulation in the intervillous space [7]. When this invasion process is compromised, various types of placental lesions can occur, which impair maternal-fetal exchanges and can manifest in pregnancy complications [8]. Both PE and FGR present histological alterations of the placenta which, although in part are different from each other, are acuminated by ischemic damage [4,5,9,10]. In placental ischemia, there is an alteration of the balance between reactive oxygen species (ROS), including superoxide anion (O2-) and hydrogen peroxide (H2O2) and antioxidants. This event is the cause of oxidative stress, which causes damage to proteins, lipids, and DNA [3,4,5,11,12]. In this way, large amounts of ROS are formed in cell membranes, endoplasmic reticulum, and mitochondria [11]. The antioxidant factors produced by the placenta and the nitric oxide (NO) vasodilator are unable to compensate for this alteration, which can lead to some serious complications [11]. The relative role of maternal hemodynamics [13,14], of patterns of fetal growth [15,16], and placental histology [17,18,19] in relation to HDP, FGR, or both [20,21,22] still need to be elucidated, as it is currently difficult to develop therapeutic or preventive strategies [5].

Moreover, recent research findings pointed out suboptimal maternal diet [23,24,25] and maternal stress as potentially important contributors [26]. We hypothesize that the analysis of the placental histology might contribute to differentiate the phenotypes of HDP and of FGR as well as their possible overlapping in the real-life clinical scenario. The purpose of this study is to describe and analyze placental histological characteristics and their possible association with different phenotypes of hypertensive disorders of pregnancy, fetal growth restriction, and in uneventful pregnancies. This association could help to design future preventive and therapeutic strategies.

## 2. Materials and Methods

### 2.1. Study Design and Participants

This is a case-control study performed at the high-risk clinic of the Maternal-Fetal Medicine Unit, tertiary referral center, University of Milan. The Ethical Committee approved the study (Ethical Committee NN Milano 10818).

All admitted singleton pregnancies affected by HDP and/or FGR who signed the research consent were included in the study. Controls were enrolled from the low-risk [27] outpatient clinic when a case was recruited and followed until delivery. Participants were eligible for the analysis only if a complete follow-up of pregnancy outcome was available.

HDP was diagnosed and classified according to the Canadian Guidelines from the Society of Obstetrics and Gynaecology of Canada (SOGC) [16], with the exclusion of chronic hypertension. Gestational age was determined by the fetal crown–rump length (CRL) [28], and fetal growth was assessed on the recorded measurements obtained by routine ultrasound measurements.

FGR was defined when the abdominal circumference (AC) was ≤10th percentile for local standards or crossed more than 2 quartiles on growth charts from the mid-trimester scan. Early and late FGR were defined according to the Delphi criteria [29]. Fetuses with an AC >10th percentile and normal uterine and umbilical Doppler velocimetry were diagnosed as having an appropriate fetal growth (AGA fetus).

Exclusion criteria included: maternal age <18 years, pregnant women with fetuses affected by chromosomal abnormalities, multiple pregnancies, congenital infections or malformations, and refusal to participate.

We excluded pregnant women who had possible comorbidities that could influence the development of the placenta and with direct and indirect risk factors, such as chronic hypertension, pre-existing diabetes, or congenital and/or acquired thrombophilia, and pregnancies conceived by in vitro fertilization.

Patients who agreed to participate in the study and who did not meet exclusion criteria were divided into the following groups based on clinical and ultrasound characteristics: (1) pregnancies complicated by HDP and associated with AGA fetus (HDP-AGA fetus); (2) pregnancies complicated by HDP and associated with FGR (HDP-FGR); (3) normotensive patients with early (gestational age ≤ 32 weeks) FGR (early FGR); (4) normotensive patients with late (gestational age > 32 weeks) FGR (late FGR); and (5) uneventful control pregnancies.

Maternal, obstetric, and neonatal characteristics as well as biophysical and biochemical markers were collected. In particular, maternal characteristics included gestational age at recruitment (weeks), age (years), pre-pregnancy BMI (kg/m^2^), smoking status during pregnancy (never or current), family history of hypertension, and parity. Obstetric characteristics included gestational age at delivery (weeks), obstetric history (previous HDP or FGR), and delivery mode (spontaneous labor, induction, and caesarean section). Neonatal characteristics included weight percentile, neonatal intensive care unit (NICU) admission, perinatal morbidity (at least one of the following: jaundice, anemia, hypoglycemia, hypocalcemia, polycythemia, respiratory distress syndrome, patent ductus arteriosus, gastro-esophageal reflux, acute kidney insufficiency, neonatal hemolysis, pneumothorax, necrotizing enterocolitis, infections, and hypoxic-ischemic encephalopathy), and neonatal death. Biophysical markers measurements included fetal biometry and Doppler interrogation of the uterine (UtA) and umbilical arteries (UA) and middle cerebral artery (MCA).

### 2.2. Assessment of Biochemical Marker

At recruitment, the concentration of placental vascular growth factors (PlGF) and soluble blocking factors (sFlt-1) was measured. Blood samples were collected in a 3.5 mL test tube containing acrylic gel. Blood samples were then centrifuged to obtain the serum and stored at −80 degrees. The samples were defrosted within 6 months of collection (to avoid serum deterioration), and sFlt-1 and PlGF concentrations were measured using the automatic Elecsys immunoassays (ROCHE), technology based on an automated immunofluorescence concentration and an ELISA immunoassay. The clinical staff was blinded to biomarkers results.

### 2.3. Placental Examination

The macroscopic analysis of the placenta included an extensive evaluation of the maternal and fetal plates, the umbilical cord, and membranes. Placental was evaluated in a blinded fashion using the Amsterdam Placental Workshop Group Consensus criteria [10]. According to standard international methods [10], at least five blocks of tissue samples were obtained for each placenta: one roll of the free membrane (chorion and amnion with attached decidua capsularis), one block containing 3 cross-sections of the umbilical cord (one from the fetal end, one near the placental insertion of the cord, and one midway between the fetal and placental ends of the cord), and three blocks containing full-thickness sections of grossly normal placental parenchyma (one at the center close to the umbilical cord insertion, one at the margin, and one midway between these two). If present, all macroscopic lesions have been described, and a block of grossly identified lesion was sampled as well, with the adjacent normal parenchyma.

Formalin-fixed, paraffin-embedded placental tissue samples were processed for conventional histopathological examination, and standard 4 mm thick tissue sections, stained with hematoxylin and eosin, were examined by light microscopy.

Placental histology was analyzed by a senior dedicated pathologist (G.B.). The pathologist was blinded to clinical characteristics except for the gestational age at delivery since some of the pathological findings are closely dependent on gestational age (i.e., maturation of placental parenchyma). Other clinical parameters were not provided to the pathologist.

### 2.4. Statistical Analysis

Categorical variables were expressed as absolute frequencies (n) and proportions (%), whereas continuous variables were expressed as means ± standard deviations (SD). We evaluated differences among proportions and means using the Fisher’s exact test and ANOVA, respectively. Odds ratios (ORs) and corresponding 95% confidence intervals (CIs) of the risk of placental injury according to pregnancy complication groups were estimated by means unadjusted and adjusted logistic regression models. The adjusted models also included terms for maternal age (years, in continuous), pre-pregnancy BMI (kg/m^2^, in continuous), smoking status (never vs. current during pregnancy), gestational age at delivery (weeks, in continuous), soluble fms-like tirosin-kinasi 1 to placental growth factor ratio (in continuous), placental weight (g, in continuous), and placental area (cm^2^, in continuous). All statistical tests were two-sided with a significance level set at <0.05. All analyses were conducted using R version 4.0.5. (R Core Team, 2021)

## 3. Results

About 600 women come to our institution for high-risk antenatal care each year. The study was conducted from 1st May 2019 to 31st December 2021. In this period of time, out of 1876 single pregnancies evaluated in our clinics, a total of 72 (3.8%) patients met the inclusion criteria and agreed to participate in the study. At the same time, 16 uneventful pregnancies were recruited as controls, for a total of 88 participants.

We divided patients into five groups according to maternal and fetal characteristics: (1) *n* = 10 pregnancies complicated by HDP and associated with AGA fetus (HDP-AGA fetus); (2) *n* = 26 pregnancies complicated by HDP and associated with FGR (HDP-FGR); (3) *n* = 12 normotensive patients with early (gestational age ≤ 32 weeks) FGR (early FGR); (4) *n* = 24 normotensive patients with late (gestational age > 32 weeks) FGR (late FGR); and (5) *n* = 16 uneventful control pregnancies. Table 1 reports maternal, obstetric, and neonatal characteristics according to pregnancy complication groups and controls.

Table 2 shows biophysical and biochemical markers.

The early FGR group reported significantly higher uterine pulsatility index (1.62 ± 0.54; *p* < 0.01) and umbilical artery pulsatility index (1.73 ± 0.33; *p* < 0.01) compared to others groups. Conversely, the early FGR group had a significantly lower cerebral placental ratio (0.93 ± 0.27; *p* < 0.01). The HDP-FGR group showed significantly higher level of sFlt-1 (8916.52 ± 7500.87 pg/ML; *p* < 0.01) and significantly lower level of PlGF (39.35 ± 50.62 pg/ML; *p* < 0.01). According to previous results, the ratio between sFlt-1 and PlGF was significantly higher (584.37 ± 798.73) in the HDP-FGR group.

Table 3 reports placental characteristics according to Amsterdam classification system of placental pathology.

The early FGR group showed a significantly lower placental weight (214 ± 84 g; *p* < 0.01) and area (409 ± 142 cm^2^; *p* < 0.01) compared to all other groups. The maternal vascular malperfusion was significantly higher in the HDP-FGR group (92%; *p* < 0.01). This result was mainly driven by placental infarction, distal villous hypoplasia, and decidual arteriopathy. The late FGR group showed higher proportion of delayed villous maturation (83%; *p* = 0.01). Of interest, this high proportion was significantly different from what was observed in early FGR.

Table 4 reports the ORs and 95% CIs of the risk of placental lesions according to pregnancy complication groups and controls.

The unadjusted models showed a significantly higher risk of vascular maternal malperfusion for the HDP-FGR group (OR = 20.00; 95% CI: 4.03–155.90) and for early FGR group (OR = 8.33; 95% CI: 1.55–67.77) than controls. The adjusted model showed a significantly higher risk for HDP-FGR group (OR = 54.94; 95% CI: 2.90–2902.41). The risk of delayed villous maturation was significantly higher in the late FGR group compared to controls in both unadjusted (OR = 8.33; 95% CI: 2.04–40.73) and adjusted (OR = 21.64; 95% CI: 3.54–171.87) analyses. The HDP-FGR group showed a higher risk of delayed villous maturation in the adjusted analyses (OR = 16.83; 95% CI: 1.85–204.44).

## 4. Discussion

Our findings suggest that maternal vascular malperfusion of the placental bed affected more than half pregnant women in every groups, but it was significantly more observed in placenta of pregnant women affected by HDP-FGR (92%) and early-FGR (83%) than in HDP-AGA fetus (60%), late FGR (63%), and even uneventful control pregnancies (38%). These two groups also showed significantly higher values of the uterine and umbilical arteries Doppler velocimetry and of the sFlt-1/PLGF ratio, the latter being a marker of the oxidative stress of the syncytiotrophoblast.

These findings demonstrate that the severe complications that these pregnancies develop both on the fetal side (fetal growth restriction) and then on the maternal side (hypertension) are a consequence of an early shallow trophoblastic invasion, poor placental development, poor fetal nutrition, and eventually placental oxidative stress. As expected, pregnancies affected by HDP-FGR had the worst obstetric outcome with the lowest gestational age at delivery and the highest incidence of cesarean sections and neonatal deaths.

Opposite to these findings, lesions consistent with delayed villous maturation were significantly more common in women with pregnancies complicated by late FGR (83%).

The decidual arteriopathy was found with a significant prevalence both in all women with hypertensive disorders and in those with early and late FGR but was observed only in one case of the 16 uneventful pregnancies.

Our data show that maternal and fetal vascular malperfusion of the placenta are not mutually exclusive. Placental developmental biology is a complex phenomenon during which different insults might produce overlapping histological lesions, and the same disease might also produce different severity and timeframe of occurrence of the same adverse clinical outcome.

However, the different prevalence of maternal vascular malperfusion in placenta of pregnancies affected by hypertensive disorders and FGR is in agreement with previous reported pathological [30,31,32,33,34,35] and clinical findings that eventually support the different etiology of the main phenotypes of hypertensive disorders of pregnancy [3].

In agreement with previously reported data, our results show a significant association between the presence of lesion of maternal vascular malperfusion and abnormal Doppler velocimetry [34,35,36]. Of interest, an abnormal uterine PI, which is a proxy of reduced uterine blood flow volume [37], was also significantly associated with decidual arteriopathy. The abnormal uterine blood flow and the malperfusion of the placental bed might enhance oxidative stress damage, inducing additional placental hypo-perfusion, resulting in turn to distal villous hypoplasia and infarction [38], as observed in our cases. Indeed, the by-products of oxidative stress of the syncytiotrophoblast generate an abnormal concentration PlGF and sFlt-1 and reverberate on the maternal endothelium, causing vasoconstriction and maternal hypertension [39].

The late FGR group has a relatively good obstetric and neonatal outcome with an adequate gestational period for delivery, a small number of caesarean sections, and no neonatal deaths. In these cases, the placental lesions consistent mainly with delayed villous maturation (83%). This type of histological lesion of the placenta represents a “maldevelopment” of the villous tree. According to the Amsterdam criteria [10], delayed villous maturation exhibits monotonous villous population with reduced numbers of vasculosyncytial membranes for the gestational age as well as a continuous cytotrophoblast layer and centrally placed capillaries. This finding appears late in pregnancy [10]. The delayed occurrence of villous maturation might be related to a hypoxic-oxidative environment that manifests late in pregnancy, likely when placental growth reaches its functional limits [40]. However, the severity of these late maldevelopments is not enough to produce high values of sFlt-1/PlGF ratio, a marker of oxidative stress.

Many preventive strategies have been proposed for the prevention of these pregnancy complications, such as angiogenic factors, nitric oxide (NO) and L-arginine supplementation, endothelial stressor inhibitors, H2S donors, statins, and antioxidants [41]. However, so far, the only preventive intervention supported by robust evidence is the administration of low-dose aspirin since early gestion in women who proved at risk of preeclampsia at the first-trimester screening [42].

However, this preventive strategy is poorly effective in HDP occurring late in gestation and frequently associated with normally grown fetuses or late FGR [43]. This limitation is likely to be the consequence of the assumption that HDP and preeclampsia are not a syndrome and that one preventive intervention fits for all phenotypes. Indeed, “maternal syndrome preeclampsia”, as associated with obesity and metabolic syndrome, is now acknowledged as one of the main causative risk factors and predisposing conditions for hypertensive disorders of pregnancy [44].

If obesity and metabolic syndrome is the villain of HDP with normally grown fetuses and late IUGR, and low-dose aspirin is partially ineffective to prevent this condition, then a different preventive measure might be enforced in these pregnant women.

Growing evidence has shown the positive effects of the Mediterranean diet (MD) on health outcomes (i.e., lower incidence of cardiovascular and metabolic diseases, lower incidence of neurodegenerative diseases, lower incidence of multiple cancer sites, and lower mortality) [23,24,25,26,45]. The traditional Mediterranean diet is a plant-oriented dietary pattern characterized by a high intake of vegetables, fruit, whole grains, legumes, and nuts; a moderate intake of dairy products (mostly cheese and yoghurt); a moderate intake of fish and poultry; a low intake of red meat; a high intake of extra-virgin olive oils, used as the main source of fat; and a moderate intake of wine, consumed with meals [46]. Maternal adherence to MD may play a role in placental development and function formation. Indeed, B vitamins act as substrates in different cellular pathways and play a role as cofactors in events such as cell multiplication, apoptosis, and intracellular signaling, all of which can target oxidative damage [47,48]. These processes are also affected by saturated fats, trans fatty acids, and cholesterol [49]. For this reason, the adherence to a diet rich in B vitamins and monounsaturated and polyunsaturated fats may positively affect the placenta and pregnancy outcome [25,50]. In addition, a diet rich in vegetables, fruits, poultry, and fish was associated with a 25% reduction in the risk of FGR [51].

Furthermore, pregnant women who had a low adherence to the MD showed smaller placentas and with a greater vascular resistance than pregnant women who highly adhered to the MD [52,53]. The placentation of women with low MD adherence was characterized by pathological vascular remodeling, increased inflammation, oxidative stress, and rapid cell division that caused similar lesions as described in our study [52,53,54].

Moreover, the MD reduces the circulation of inflammatory markers and endothelial dysfunction, such as C-reactive protein and E-selectin [55,56,57].

Finally, recent epigenetic studies have investigated the role of MD in the early stages of embryonic growth, demonstrating how correct nutrition plays a fundamental role already in the periconceptional period [58].

One of the strengths of this study is that we rigorously met the criteria for blinded placental pathology examination by applying a systematic and rigorous approach to classify placental characteristics, based on an international consensus. This approach avoids the limitations on local policies and definitions. The consecutive recruitment foreseen according to the design of the study was partially interrupted by the first two waves of the COVID-19 pandemic, when our high-risk maternity was designed as the main regional hub in Lombardy for SARS-COV-2 infections. Furthermore, our controls and cases are not matched for gestational age at delivery. This aspect should be further investigated, but as such, this would introduce further limitations due to the different etiology of premature delivery that, obviously, could not be considered as “normal” controls. A small sample of patients and the lack of information on patients by patient dietary status are also additional limiting factors of the study.

In conclusion, this study suggests a similar, significantly higher prevalence of placental maternal vascular malperfusion in HDP-FGR and early FGR. This might imply a common etiology. Conversely, HDP-AGA fetus, which includes preeclampsia not associated with FGR, and late FGR represent a more heterogeneous condition mainly but not always characterized by abnormal villous tree development and maturation. The different prevalence of patterns of placental lesions were coherently associated with indices of feto-placental Doppler interrogation and sFlt-1/PlGF ratio. These conditions are likely associated with different etiologies, such as maternal pre-pregnancy risk factors for metabolic syndrome. These findings suggest a possible preventive nutritional approach in addition to low-dose aspirin for pregnant women with predisposing factors for HDP-AGA fetuses and late FGR.

## Figures and Tables

**Table 1:** Maternal, obstetric, and neonatal characteristics ^a^ according to pregnancy complication groups and controls.

Variable	Pregnancy Complication Group					*p*-Value
	HDP-AGA Fetus (*n* = 10)	HDP-FGR (*n* = 26)	Early FGR (*n* = 12)	Late FGR (*n* = 24)	Controls (*n* = 16)	
Maternal characteristics						
Gestational age at recruitment (weeks)	36.8 ± 2.4	31.1 ± 3.9	28.0 ± 3.6	34.1 ± 4.1	32.7 ± 5.0	*p* < 0.01 ^b^
Age (years)	34 ± 4	34 ± 6	33 ± 6	30 ± 6	35 ± 3	*p* = 0.13 ^b^
Pre-pregnancy BMI (kg/m^2^)	25.9 ± 4.9	24.5 ± 5.8	22.1 ± 3.3	21.7 ± 3.2	22.3 ± 3.8	*p* = 0.05 ^b^
Smoking status during pregnancy	0 (0)	1 (4)	4 (33)	4 (17)	0 (0)	*p* = 0.02 ^c^
Family history of hypertension	7 (70)	16 (62)	5 (42)	14 (58)	10 (63)	*p* = 0.74 ^c^
Nulliparous women	8 (80)	19 (73)	5 (42)	18 (75)	11 (69)	*p* = 0.29 ^c^
Obstetric characteristics						
Gestational age at delivery (weeks)	38.0 ± 2.4	32.8 ± 3.9	33.2 ± 3.9	38.5 ± 1.8	40.0 ± 1.3	*p* < 0.01 ^b^
Obstetric history, previous HDP or FGR	2 (20)	5 (19)	3 (25)	1 (4)	0 (0)	*p* = 0.08 ^c^
Caesarean section	5 (50)	25 (96)	10 (83)	5 (21)	2 (13)	*p* < 0.01 ^c^
Neonatal characteristics						
Neonatal weight percentile	54 ± 30	6 ± 3	2 ± 3	5 ± 3	49 ± 18	*p* < 0.01 ^b^
NICU admission ^d^	1 (10)	17 (65)	9 (75)	3 (13)	0 (0)	*p* < 0.01 ^c^
Perinatal morbidity	1 (10)	12 (46)	8 (67)	1 (4)	0 (0)	*p* < 0.01 ^c^
Neonatal death	0 (0.0)	4 ^e^ (15)	1 ^f^ (8)	0 (0.0)	0 (0)	*p* = 0.11 ^c^
Time recruitment-to-delivery (weeks)	1.2 ± 1.2	1.8 ± 2.3	5.2 ± 2.7	4.5 ± 3.7	7.3 ± 5.2	*p* < 0.01

^a^ Continuous variables are expressed as mean ± standard deviation, and categorical variables are expressed as absolute frequencies and percentages in parenthesis; ^b^ calculated by means of ANOVA test; ^c^ calculated by means of Fisher’s exact test; ^d^ one missing value in the HDP-FGR group and in the early FGR group, respectively; ^e^ three perinatal deaths and one intrauterine fetal death; ^f^ medical termination of pregnancy. Abbreviations: BMI, body mass index; FGR, intrauterine growth restriction; HDP, hypertensive disorders of pregnancy; NICU, neonatal intensive care unit. Gestational age at recruitment was significantly lower in the early FGR group (mean ± SD: 28.0 ± 4.1 weeks; *p* < 0.01) compared to other groups. Women belonging to HDP-AGA fetus group had a significantly higher pre-pregnancy BMI (25.9 ± 4.9 kg/m^2^; *p* = 0.05), whereas women in the early FGR group were more likely to be smokers during pregnancy (33.3%) than women of other groups. Women in the HDP-FGR group had a significantly lower gestational age at delivery (32.8 ± 3.9 weeks; *p* < 0.01) and a significantly higher proportion of caesarean section (96.2%; *p* < 0.01). Neonatal weight percentile was significantly lower in the early FGR group (2 ± 3; *p* < 0.01). In addition, the early FGR group showed significantly higher proportion of NICU admission (75.0%; *p* < 0.01) and perinatal morbidity (66.7%; *p* < 0.01). No significant difference emerged for the other maternal, obstetric, and neonatal characteristic considered.

**Table 2:** Biophysical and biochemical markers ^a^ according to pregnancy complication groups and controls.

Variable	Pregnancy Complication Group					*p*-Value
	HDP-AGA Fetus (*n* = 10)	HDP-FGR (*n* = 26)	Early FGR (*n* = 12)	Late FGR (*n* = 24)	Controls (*n* = 16)	
Biophysical marker						
Uterine pulsatility index	0.74 ± 0.30	1.32 ± 0.39	1.62 ± 0.54	0.75 ± 0.22	0.70 ± 0.21	*p* < 0.01 ^b^
Umbilical artery pulsatility index	0.89 ± 0.16	1.33 ± 0.48	1.73 ± 0.33	0.95 ± 0.18	0.92 ± 0.15	*p* < 0.01 ^b^
Middle cerebral artery pulsatility index ^c^	1.75 ± 0.27	1.66 ± 0.64	1.57 ± 0.42	1.74 ± 0.41	1.82 ± 0.39	*p* = 0.69 ^b^
Cerebral placental ratio ^c^	2.06 ± 0.58	1.39 ± 0.62	0.93 ± 0.27	1.84 ± 0.34	1.99 ± 0.47	*p* < 0.01 ^b^
Biochemical marker						
Soluble fms-like tirosin-kinasi 1 (pg/ML) ^d^	5872 ± 3755	8916 ± 7500	4282 ± 3693	3247 ± 2345	2020 ± 1104	*p* < 0.01 ^b^
Placental growth factor (pg/ML) ^e^	60 ± 37	39 ± 51	155 ± 267	230 ± 261	498 ± 438	*p* < 0.01 ^b^
Soluble fms-like tirosin-kinasi 1 to placental growth factor ratio ^f^	187 ± 341	584 ± 799	129 ± 180	33 ± 39	25 ± 60	*p* < 0.01 ^b^

^a^ Expressed as mean ± standard deviation; ^b^ calculated by means of ANOVA test; ^c^ one missing value in controls; ^d^ two missing values in controls, one missing value in the HDP-AGA fetus group, and three missing values in the HDP-FGR group; ^e^ one missing value in the HDP-AGA fetus group, three missing values in the HDP-FGR group, and one missing value in the early FGR group; ^f^ two missing values in controls, one missing value in the HDP-AGA fetus group, two missing values in the HDP-FGR group, and one missing value in the early FGR group.

**Table 3:** Placental characteristics ^a^ and Amsterdam classification system of placental injury according to pregnancy complication groups and controls.

Variable	Pregnancy Complication Group					*p*-Value
	HDP-AGA Fetus (*n* = 10)	HDP-FGR (*n* = 26)	Early FGR (*n* = 12)	Late FGR (*n* = 24)	Controls (*n* = 16)	
Placental characteristics						
Placental weight (g) ^b^	512 ± 116	269 ± 114	214 ± 84	411 ± 70	502 ± 54	*p* < 0.01 ^c^
Placental area (cm^2^)	968 ± 320	505 ± 175	409 ± 142	745 ± 306	870 ± 165	*p* < 0.01 ^c^
Feto-placental ratio ^b^	6.0 ± 0.7	5.5 ± 2.7	6.0 ± 1.2	6.3 ± 1.1	6.6 ± 0.6	*p* = 0.27
Amsterdam classification system						
Maternal vascular malperfusion	6 (60)	24 (92)	10 (83)	15 (63)	6 (38)	*p* < 0.01 ^d^
*Placental infarction*	3 (30)	12 (46)	3 (25)	8 (33)	0 (0)	*p* = 0.01 ^d^
*Retroplacental hemorrhage*	0 (0)	4 (15)	2 (17)	1 (4)	0 (0)	*p* = 0.24 ^d^
*Distal villous hypoplasia*	0 (0)	11 (42)	2 (17)	3 (13)	1 (6)	*p* = 0.01 ^d^
*Accelerated villous maturation*	5 (50)	15 (58)	5 (42)	9 (38)	4 (25)	*p* = 0.30 ^d^
*Decidual arteriopathy*	3 (30)	11 (42)	8 (67)	5 (21)	1 (6)	*p* < 0.01 ^d^
Fetal vascular malperfusion	0 (0)	3 (12)	1 (8)	0 (0)	0 (0)	*p* = 0.24 ^d^
Delayed villous maturation	4 (40)	13 (50)	5 (42)	20 (83)	6 (38)	*p* = 0.01 ^d^
Villitis of unknown etiology ^e^	0 (0)	4 (15)	2 (17)	0 (0)	2 (13)	*p* = 0.23 ^d^
Ascending Intrauterine Infection	2 (20)	7 (27)	5 (42)	4 (17)	3 (19)	*p* = 0.55 ^d^

^a^ Expressed as mean ± standard deviation; ^b^ one missing value in the late FGR group; ^c^ calculated by means of ANOVA test; ^d^ calculated by means of Fisher’s exact test; ^e^ two missing values in the HDP-FGR group, and one missing value in the late FGR group.

**Table 4:** Odds ratios (OR) and corresponding 95% confidence intervals (CI) of the risk of placental injury (defined by Amsterdam classification system) according to pregnancy complication groups.

Pregnancy Complication Group	Placental Injury According to Amsterdam Classification System				
	Maternal Vascular Malperfusion	Fetal Vascular Malperfusion	Delayed Villous Maturation	Villitis of Unknown Etiology	Ascending Intrauterine Infection
	OR (95% CI)	OR (95% CI)	OR (95% CI)	OR (95% CI)	OR (95% CI)
*Unadjusted model*					
Controls	Ref.	Not applicable	Ref.	Not applicable	Ref.
HDP-AGA fetus	2.50 (0.51–13.64)		1.11 (0.21–5.68)		1.08 (0.12–8.00)
HDP-FGR	20.00 (4.03–155.90)		1.67 (0.47–6.20)		1.60 (0.37–8.47)
Early FGR	8.33 (1.55–67.77)		1.19 (0.25–5.61)		3.10 (0.58–19.09)
Late FGR	2.78 (0.77–10.80)		8.33 (2.04–40.73)		0.87 (0.16–5.00)
*Adjusted model* ^a^					
Controls	Ref.	Not applicable	Ref.	Not applicable	Ref.
HDP-AGA fetus	5.27 (0.66–50.94)		2.80 (0.36–21.97)		1.93 (0.17–20.27)
HDP-FGR	54.94 (2.90–2902.41)		16.83 (1.85–204.44)		6.31 (0.58–83.89)
Early FGR	6.54 (0.29–191.19)		7.54 (0.57–114.87)		5.06 (0.31–93.57)
Late FGR	2.45 (0.43–14.90)		21.64 (3.54–171.87)		0.88 (0.11–6.96)

^a^ Also including terms for maternal age (years), pre-pregnancy BMI (kg/m^2^), smoking status, gestational age at delivery (weeks), soluble fms-like tirosin-kinasi 1 to placental growth factor ratio, placental weight (g), and placental area (cm^2^).

## Data Availability

The data that support the findings of this study are available from the first author (D.D.D.) upon reasonable request.
